# Older Age and High α-Fetoprotein Predict Higher Risk of Hepatocellular Carcinoma in Chronic Hepatitis-B-Related Cirrhotic Patients Receiving Long-Term Nucleos(t)ide Analogue Therapy

**DOI:** 10.3390/diagnostics12092085

**Published:** 2022-08-28

**Authors:** Yuh-Ying Liu, Chih-Lang Lin, Cheng-Hao Weng, Pei-Hung Chang, Cheng-Hung Chien, Kuang-Chen Huang, Man-Chin Hua, Ching-Chih Hu

**Affiliations:** 1 Liver Research Unit, Department of Gastroenterology and Hepatology, Chang Gung Memorial Hospital, Keelung 20401, Taiwan; 2 College of Medicine, Chang Gung University, Taoyuan 33302, Taiwan; 3 Kidney Research Center, Department of Nephrology, Linkou Chang Gung Memorial Hospital, Taoyuan 333, Taiwan; 4 Department of Oncology, Chang Gung Memorial Hospital, Keelung 20401, Taiwan; 5 Department of Pediatrics, Chang Gung Memorial Hospital, Keelung, 20401, Taiwan

**Keywords:** chronic hepatitis B, cirrhosis, cumulative incidence, incidence rate, risk, hepatocellular carcinoma, tenofovir, entecavir

## Abstract

Background: Nucleos(t)ide analogues (NUCs) were proved to reduce hepatocellular carcinoma (HCC) development in chronic hepatitis B (CHB) patients, but data were limited on their efficacy in cirrhotic CHB patients. Methods: A total of 447 cirrhotic CHB patients treated with tenofovir/entecavir were retrospectively analyzed and divided into HCC (*n* = 48) and non-HCC (*n* = 399) groups. The median follow-up period was 62.1 months. Results: A total of 48 patients (10.7%) developed HCC during surveillance. The annual incidence rate of HCC was 2.04 per 100 person-years. The cumulative incidence of HCC was 0.9%, 9.8%, and 22.1% at 1, 5, and 10 years, respectively. Significant predictors for HCC identified using a multiple Cox regression analysis were age ≥50 years (hazard ratio (HR): 2.34) and α-fetoprotein (AFP) ≥8 ng/mL (HR: 2.05). The incidence rate of HCC was 8.67-fold higher in patients with age ≥50 years and AFP ≥8 ng/mL (3.14 per 100 person-years) than those with age <50 years and AFP <8 ng/mL (0.36 per 100 person-years). Conclusions: Cirrhotic CHB patients with age <50 years and AFP <8 ng/mL had the lowest annual incidence of HCC. However, those with age ≥50 years or/and AFP ≥8 ng/mL had a significantly higher risk for HCC development and warrant a careful surveillance schedule.

## 1. Introduction

Hepatocellular carcinoma (HCC), which accounts for most liver cancers, is the sixth most common cancer in the world. It is also the third-leading cause of cancer-related mortality, causing more than 800,000 deaths per year [1,2]. Chronic hepatitis B virus (HBV) infection causes global health problems; more than 240 million people have the disease. Without treatment, 40% of chronically infected patients will progress to cirrhosis, which increases the risk of HCC [3]. Chronic HBV infection is also the most common cause of HCC and is associated with more than 50% of HCC cases [4,5]. Long-term therapy with nucleos(t)ide analogues (NUCs) has been well demonstrated to result in improvement of liver necroinflammation and fibrosis, as well as regression of cirrhosis [6,7,8,9]. The risk of HCC development was also reported to be reduced significantly in the NUC-treated patients with advanced fibrosis or cirrhosis. The risk reduction was more prominent in patients with maintained viral suppression than in those with a virological breakthrough [10,11,12]. Due to a low drug-resistance rate and a high potency of viral suppression, entecavir (ETV) and tenofovir disoproxil fumarate (TDF) have become the first-line NUC regimen for CHB treatment. Previous studies showed that antiviral therapy with ETV reduced the risk of HCC in cirrhotic patients, particularly among those with maintained viral suppression. [12]. The rate of the reduction in HCC incidence was also more in the ETV-treated than those with LAM-treated cirrhotic patients [13]. However, the risk of HCC is not completely eliminated by NUC therapy.

The long-term use of NUC therapy for cirrhotic CHB patients has been reimbursed by Taiwan’s national health insurance system since 2010. There are few studies that focused on assessing the predictors of in-treatment HCC development in CHB-related cirrhotic patients with NUC therapy. Therefore, this retrospective study was conducted to elucidate the risks and predictors of HCC development during NUC therapy and to identify high-risk patients that warrant intensive surveillance during therapy.

## 2. Materials and Methods

### 2.1. Study Population

The patients that were diagnosed with CHB-related cirrhosis and that had initiated long-term ETV monotherapy (0.5 mg daily) or TDF monotherapy (300 mg) in our liver research unit in April 2007 and August 2013 were enrolled in this study. Chronic HBV infection was defined as being seropositive for HBsAg for more than 6 months. Baseline clinical and biochemical data were recorded upon the initiation of ETV or TDF therapy. The diagnosis of liver cirrhosis was made using a liver biopsy specimen with an Ishak modified histology activity index score ≥ 5 or Metavir score = 4, or ultrasonography using the previously described cirrhosis scoring system [14] with splenomegaly or esophageal/gastric varices. Fatty liver was diagnosed based on semiquantitative assessment using ultrasound according to the presence of increased liver echogenicity to the kidney [15,16]. All patients had serum HBV DNA ≥2000 IU/mL at baseline. Patients with HCC diagnosed before and within 6 months after the beginning of therapy or with a follow-up duration of less than 6 months were excluded from the study. We also excluded CHB patients coinfected with chronic hepatitis C or human immunodeficiency virus, toxic hepatitis, autoimmune hepatitis, primary biliary cirrhosis, or Wilson’s disease. This study was approved by the Medical Ethics Committee of Chang Gung Memorial Hospital (Institutional Review Board approval number: 104_9790B & 201601917B0) and was carried out in accordance with the code of ethics of the World Medical Association (Declaration of Helsinki).

### 2.2. Follow-Up for HCC Surveillance

All patients received NUCs for the entire follow-up period and were observed from the beginning of the NUC therapy to the date of HCC diagnosis, last visit, or death. Liver ultrasonography and laboratory examination were routinely checked every three months. The diagnosis of HCC was confirmed using the histological evaluation of a needle biopsy sample or surgically resected specimens, two typical image studies such as dynamic liver computed tomography or magnetic resonance imaging, or one image study plus an increased serum AFP level of more than 400 ng/mL [17,18].

### 2.3. Statistical Analysis

After testing for normal distribution using the Kolmogorov–Smirnov test, the continuous variables were against normal distribution and were reported as the median (interquartile range). The categorial variables were summarized as a number (percentage). The differences between the continuous and categorical variables were compared using the Mann–Whitney U test, Fisher’s exact test, and the chi-squared test where appropriate. A Cox proportional hazards regression model was used to assess the clinical, biochemical, and virological variables associated with the risk of HCC development. The cumulative incidence of HCC was evaluated using the Kaplan–Meier method and compared using a log-rank test. All statistical tests were two-tailed, with *p*-values < 0.05 considered statistically significant. Data were analyzed using SPSS 23 software for Windows (SPSS, Inc., Chicago, IL, USA).

## 3. Results

### 3.1. Patient Characteristics

There was a total of 457 CHB patients with cirrhosis enrolled in our study. Six patients receiving ETV therapy, one patient receiving TDF therapy with a follow-up duration of less than 6 months, and three patients with HCC development within the first 6 months of enrollment were excluded. Finally, 447 patients were included in our final analysis. A total of 48 patients were diagnosed with HCC; 17 (35.4%) patients were confirmed using a liver biopsy, 5 (10.4%) patients were diagnosed using a serum AFP ≥400 ng/mL plus one imaging modality, and the remaining 26 patients were determined using two typical imaging modalities. The study patients were divided into HCC (*n* = 48) and non-HCC (*n* = 399) groups. The baseline clinical and biochemical data of the patients upon initiation of the NUC therapy are presented in Table 1. The mean age of the cirrhotic patients at initiation of the NUC therapy was 55.3 ± 11.6 years; 339 (75.8%) patients were men. The proportion of patients with age ≥50 years in the HCC and non-HCC groups were 79% and 66%, respectively. Patients with HCC development during NUC therapy had higher baseline AFP and HBV DNA levels. There was also a high percentage of patients in the HCC group that exhibited a baseline AFP level ≥8 ng/mL (HCC group vs. non-HCC group: 60% vs. 42%, respectively; *p* = 0.016).

### 3.2. Cumulative Incidence of HCC

The median follow-up period was 62.1 months (range: 6.1–144.6 months). A total of 48 patients (10.7%) developed HCC during the surveillance period, with an incidence rate of 2.04 (95% CI: 1.52–2.68) new HCC cases per 100 person-years. The cumulative incidence of HCC was 0.9%, 9.8%, and 22.1% at 1, 5, and 10 years, respectively.

### 3.3. Risk Factors Associated with HCC Development

**Figure 1:**
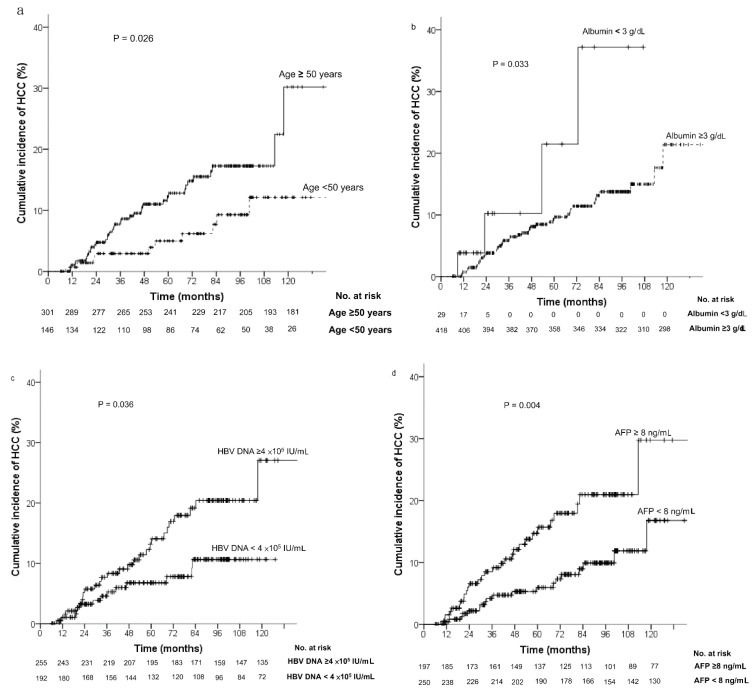

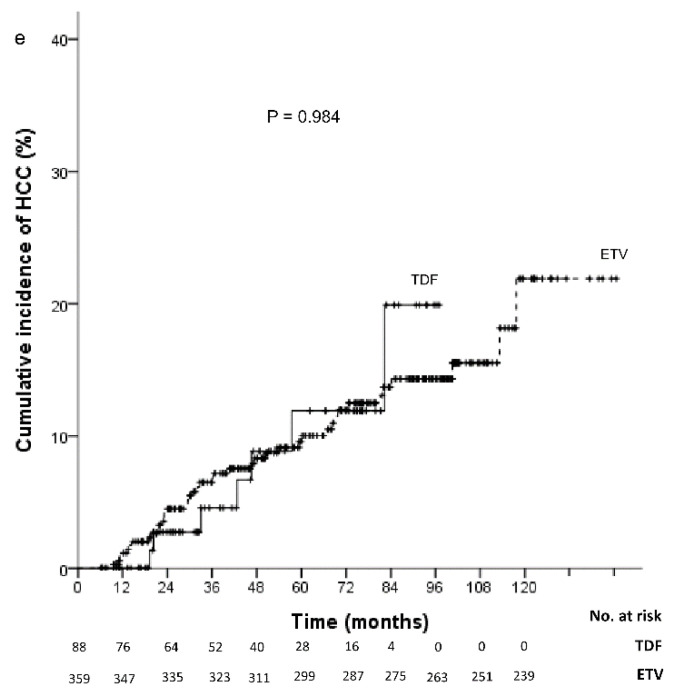
Cumulative incidence of hepatocellular carcinoma assessed using baseline risk factors: (**a**) age; (**b**) albumin; (**c**) HBV DNA; (**d**) AFP; (**e**) NUCs. Patients with older age, higher AFP and HBV DNA levels, and low albumin levels had a higher risk for HCC development during NUC therapy. The risk of HCC development was not different between the two NUCs. AFP, α-fetoprotein; NUC, nucleos(t)ide analogue; TDF, tenofovir; ETV, entecavir.

Factors associated with the risk of HCC development were assessed using the Kaplan–Meier method and compared using the log-rank test (Figure 1). Patients with HCC development during NUC therapy were significantly older (age ≥50 years) (*p* = 0.026), had higher pretreatment serum HBV DNA levels (≥4 × 10^5^ IU/mL) (*p* = 0.036) and AFP levels (≥8 ng/mL) (*p* = 0.004), and had lower albumin levels (<3 g/dL) (*p* = 0.033). However, different NUCs did not affect the risk of HCC development.

Baseline clinical and biochemical factors associated with HCC development, including age ≥50 years (hazard ratio (HR): 2.17, 95% confidence interval (CI) = 1.08–4.36), serum HBV DNA levels ≥4 × 10^5^ IU/mL (HR: 1.96, 95% CI = 1.03–3.74), albumin <3 g/dL (HR: 2.91, 95% CI = 1.04–8.15), and AFP ≥8 ng/mL (HR: 2.29, 95% CI = 1.28–4.1), were identified using a univariate Cox regression analysis. These significant factors were then entered into a stepwise multiple regression analysis. The results of the multivariate Cox regression analysis showed a treatment age ≥50 years (HR: 2.34, 95% CI = 1.08–5.1) and an AFP ≥8 ng/mL (HR: 2.05, 95% CI = 1.1–3.84) were significant independent predictors of HCC development during NUC treatment (Table 2).

### 3.4. Incidence of HCC According to Risk Factors

**Figure 2:**
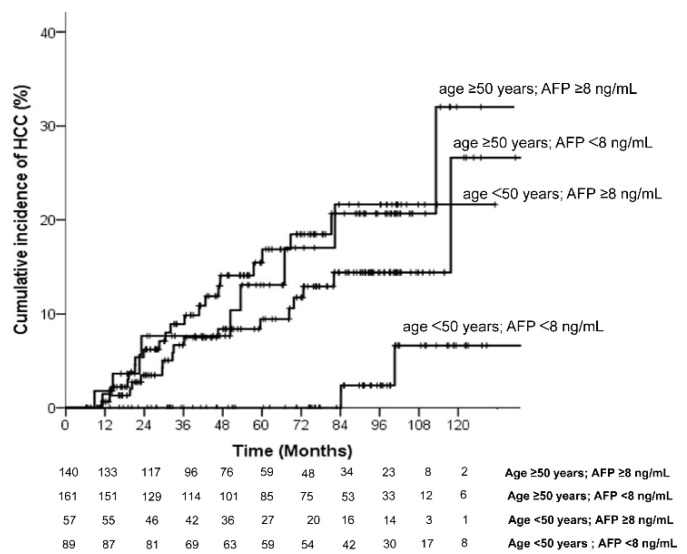
Cumulative incidence of hepatocellular carcinoma according to age and AFP.

The analysis of the incidence rate of HCC development was then stratified into subgroups according to the risk factors identified by the multivariate analysis (Table 3). As compared to the patients with treatment age <50 years and AFP <8 ng/mL (0.36, 95% CI = 0.06–1.19), the incidence rates of HCC per 100 person-years were significantly higher in patients with age ≥50 years and AFP ≥8 ng/mL (3.14, 95% CI = 1.99–4.72), *p* = 0.004. The patients with age ≥50 years and AFP ≥8 ng/mL had an 8.67-fold higher rate of HCC than those with age <50 years and AFP <8 ng/mL. Among patients with either age ≥50 years or AFP ≥8 ng/mL, the incidence rates of HCC were also higher than those with age <50 years and AFP <8 ng/mL. The cumulative HCC incidence differed significantly in patients with age <50 years and AFP <8 ng/mL versus age ≥50 years and AFP ≥8 ng/mL. As shown in Figure 2, the 5-year and 10-year cumulative incidence of HCC development were 0% and 6.6%, respectively, in patients with age <50 years and AFP <8 ng/mL; and 15.4% and 32%, respectively, in patients with age ≥50 years and AFP ≥8 ng/mL.

**Figure 3:**
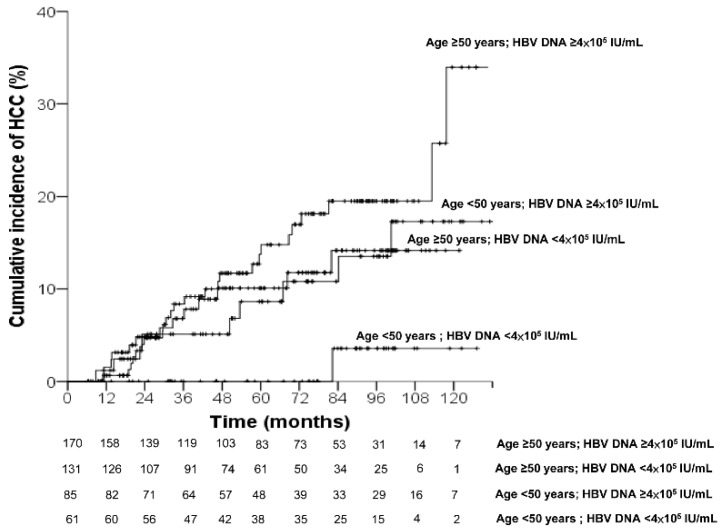
Cumulative incidence of hepatocellular carcinoma according to age and HBV DNA.

A serum HBV DNA level ≥4 × 10^5^ IU/mL was shown to be a factor associated with HCC development in univariate analysis. The HCC incidence rate was analyzed in subgroups according to age and HBV DNA levels (Table 4). As compared to the patients with treatment age <50 years and HBV DNA <4 × 10^5^ IU/mL (0.28, 95% CI = 0.01–1.39), the incidence rates of HCC per 100 person-years were significantly higher in patients with age ≥50 years and HBV DNA ≥4 × 10^5^ IU/mL (2.86, 95% CI = 1.89–4.16) (*p* = 0.024). The patients with age ≥50 years and HBV DNA ≥4 × 10^5^ IU/mL had a 9.9-fold higher rate of HCC than those with age <50 years and HBV DNA <4 × 10^5^ IU/mL. However, the HCC incidence was similar between patients with age <50 years and HBV DNA <4 × 10^5^ IU/mL and patients age ≥50 years and HBV DNA <4 × 10^5^ IU/mL or age <50 years and HBV DNA ≥4 × 10^5^ IU/mL. The cumulative HCC incidence was significantly lower in patients with age <50 years and HBV DNA <4 × 10^5^ IU/mL versus the patients in the other subgroups. As shown in Figure 3, the 5-year and 10-year cumulative incidence of HCC development were 0% and 3.6%, respectively, in patients with age <50 years and HBV DNA <4 × 10^5^ IU/mL; and 13.7% and 34%, respectively, in patients with age ≥50 years and HBV DNA ≥4 × 10^5^ IU/mL.

## 4. Discussion

In the present study, we demonstrated that long-term NUC therapy could not fully eliminate the risk of HCC development in CHB-related cirrhotic patients. Age and AFP were identified to be predictors associated with HCC development. By identifying patients with predictors for HCC development, a more intensive surveillance schedule with a shorter interval could be arranged in order to detect HCC at an early stage. In previous studies, the cumulative incidence of HCC at year 5 was 7–18% in NUC-treated cirrhotic patients [12,13,19,20,21]. Our data showed similar results; the 5-year cumulative incidence of HCC was 9.8% with an annual incidence of 2.04 per 100 person-years.

The risk factors associated with HCC development in CHB-related cirrhotic patients under NUC therapy, including older age, male sex, HBeAg positivity, statin use, platelet count, AFP and hemoglobin levels, variceal bleeding history, and 1-year virological response, had been reported in previous studies [22,23,24]. Our study demonstrated that only an older age (≥50 years) and AFP ≥8 ng/mL were predictors for risk of HCC in those patients with long-term NUC treatment. Previous reports indicated that the risk of HCC in cirrhotic patients under NUC therapy was age-dependent [22,23,24]. Tsai et al. proved that a higher risk for HCC development manifested at age 60 or higher. In our study, we actually showed that at a younger age, those ≥50 years were already predisposed to the development of HCC. The annual incidence of HCC in patients with age ≥50 years (2.04, 95% CI = 1.52–2.68) was significantly higher than those with age <50 years (1.19, 95% CI = 0.6–2.12) (per 100 person-years) (*p* = 0.037). 

Serum AFP was determined to be a serological biomarker for detection of HCC; therefore, it is commonly used for HCC surveillance [25,26]. However, an elevation in AFP levels also occurred during hepatic bridging necrosis in CHB [27]. A meta-analysis study by Zhang et al. showed that using an AFP level with a cutoff point of 400 ng/mL could detect HCC with a sensitivity of 0.32 and a specificity of 0.99 [18]. In the present study, only five (10.4%) patients with HCC development had an elevation in their AFP level of more than 400 ng/mL when HCC was detected. Previous studies reported that 40–47% of patients with HCC had normal AFP levels when the diagnosis was confirmed [28,29,30]. These HCC patients with a normal AFP were older and predominantly male. There was also a lower rate of chronic HBV infection in HCC patients with a normal AFP than in patients with an abnormal AFP level [28]. An AFP with a cutoff point of 20 ng/mL was shown to detect HCC with a sensitivity of 41–64% and a specificity of 80–94% [31,32]. The American Association for the Study of Liver Disease also recommended that HCC surveillance should be considered positive when the AFP level is greater than 20 ng/mL. In our study, 26 (54%) patients had a serum AFP level less than 20 ng/mL when HCC was detected. However, there were no significant differences in age and sex between patients with and without a normal AFP level. In addition, our study showed that a higher baseline AFP level was associated with an increased risk for HCC development in cirrhotic CHB patients during NUC therapy. The incidence rates of new HCC case per 100 person-years were significantly higher in patients with AFP ≥8 ng/mL (3.03, 95% CI = 2.07–4.3) than in those with AFP <8 ng/mL (1.36, 95% CI = 0.84–2.09) (*p* = 0.007). The patients with AFP ≥8 ng/mL had a 2.19-fold higher rate of HCC than those with AFP <8 ng/mL. It may be that an elevation in the baseline AFP is related to hepatic necroinflammation and hepatocyte proliferation, and thus plays a role in hepatocarcinogenesis.

For further analysis of the risk for HCC affected by the predictors, we stratified the patients into four groups according to the predictors identified by the multivariate analysis. The cumulative incidence of HCC in patients with age <50 years and AFP <8 ng/mL at 5 years and 10 years was 0% and 6.6%, respectively. The incidence rate of HCC of these patients was 0.36 (95% CI = 0.06–1.19) per 100 person-years. It was lower than the pooled rate of HCC incidence in CHB patients with Child–Turcotte–Pugh A cirrhosis demonstrated by a previous meta-analysis study [33]. Although the risk of HCC development in CHB-related cirrhotic patients could not be completely eliminated by long-term NUC therapy, we found this subgroup of patients obtained more benefits from therapy and had a significantly lower rate of HCC than the other subgroup of patients. On the contrary, patients with age ≥50 years or/and AFP ≥8 ng/mL had a significantly higher risk of HCC compared with those with age <50 years and AFP <8 ng/mL; therefore, these patients should be closely monitored for HCC occurrence during NUC therapy. According to the current clinical practice guidelines, HCC surveillance with a 6-month internal was recommended [5,17]. However, a meta-analysis performed by Nathani et al. demonstrated a pooled tumor volume-doubling time of 4.6 months, while 35% of the patients had a tumor volume-doubling time of less than 3 months [34]. Chronic HBV infection has been identified as a factor associated with a rapid tumor growth pattern [34,35]. In accordance with above results, we suggested the surveillance of the HBV-cirrhotic patients with ≥50 years and AFP ≥8 ng/mL could be scheduled at 3-month intervals.

The impact of male sex on HCC prediction in chronic hepatitis B patients treated with NUCs was controversial [22,23,36,37,38,39]. In the study of Papatheodoridis et al., the patients enrolled were Caucasian and male sex was proved to be a predictor of HCC in two of five models [36]. However, two studies from Asia failed to demonstrated sex as a significant predictor for HCC [37,38]. Another two studies in Taiwan that enrolled only cirrhotic HBV patients with long-term NUC therapy demonstrated that male sex was a predictor of HCC [22,39]. However, male sex was shown to not be associated with HCC in the univariate analysis and was then excluded in the multiple Cox regression model in the present study. Further studies to verify the impact of sex on HCC risk in cirrhotic HBV patients with long-term NUC therapy are needed. Previous studies that reported effective ETV and TDF treatments for HCC prevention in cirrhotic CHB patients were contentious. Most of the studies were conducted in Asia, and some reports resulted from subgroup analyses. Previous meta-analysis studies demonstrated that TDF treatment resulted in a significant lower HCC incidence than ETV treatment among cirrhosis patients [40,41]. However, yet other studies reported contrasting results. Papatheodoridis et al. reported that the hazards of HCC were similar between ETV- and TDF-treated Caucasian patients [36]. Chen et al. showed that TDF treatment was associated with a lower risk for HCC than ETV treatment. However, among patients with compensated cirrhosis or patients enrolled after 2011, the difference between the two NUCs seemed to disappear [39]. Huang et al. also demonstrated that the cumulative incidence of HCC in TDF- and ETV-treated compensated cirrhotic patients was not significantly different after propensity-score matching. The lower HCC incidence in the TDF group compared to the ETV group was only detected in patients with a high HCC risk score [42]. As shown in Figure 1e, our data showed that the incidence of HCC in cirrhotic CHB patients was not significantly different between patients treated with either of the two NUCs. A future study on HCC prevention using ETV and TDF treatment in cirrhotic patients according to the HCC risk score may elucidate which antiviral regimen is more beneficial to cirrhotic patients.

There was limitation of our study. The mean duration of the follow-up was 47.4 ± 26.5 months in TDF-treated patients and 67.0 ± 34.8 months in ETV-treated patients. Since the duration was relatively shorter in the TDF group, this warrants a longer follow-up period in a further study to clarify the long-term effects against HCC development.

## 5. Conclusions

Long-term NUC therapy could not completely eliminate the risk for HCC development in CHB-related cirrhotic patients. Cirrhotic CHB patients with age <50 years and AFP <8 ng/mL had the lowest annual incidence of HCC, which was lower than the pooled incidence of HCC in CHB patients. However, patients with age ≥50 years or/and AFP ≥8 ng/mL had a significantly higher risk for HCC and thus warrant a careful surveillance schedule.

## Figures and Tables

**Table 1:** Baseline characteristics of cirrhotic chronic hepatitis B patients with and without hepatocellular carcinoma.

Baseline Parameters	HCC (*n* = 48)	Non-HCC (*n* = 399)	*p*-Value
Age (years) ^1^	56.0 (50.3–62.0)	55.0 (47.0–63.0)	0.327
Sex (male) ^2^	40 (83)	299 (75)	0.327
Body mass index (kg/m^2^) ^1^	23.0 (20.9–27.3)	24.9 (22.6–27.8)	0.076
HBeAg (positive) ^2^	8 (16.7)	48 (12)	0.841
Albumin (g/dL) ^1^	3.9 (3.6–4.1)	3.9 (3.6–4.3)	0.341
AST (U/L) ^1^	59 (42–86)	53 (38–91)	0.494
ALT (U/L) ^1^	59 (36–106)	54 (37–102)	0.816
Total bilirubin (mg/dL) ^1^	1.3 (0.9–1.7)	1.1 (0.8–1.4)	0.239
AFP (ng/mL) ^1^	10.8 (5.0–18.6)	6.5 (4.0–15.2)	0.042
HBV DNA (log_10_ IU/mL) ^1^	6.0 (4.8–7.2)	5.5 (4.5–6.7)	0.039
White blood cells (×10^3^/μL) ^1^	6.2 (4.5–6.6)	6.2 (4.8–6.7)	0.731
Hemoglobin level (g/dL) ^1^	13.5 (12–15.2)	13.6 (11.3–14.9)	0.991
Platelet count (×10^3^/μL) ^1^	138.5 (94–152.5)	148 (111–165)	0.453
Creatinine (mg/dL) ^1^	1.0 (0.7–1.4)	1.0 (0.8–1.4)	0.932
Fatty liver ^2^	11 (23)	66 (17)	0.269
Splenomegaly ^2^	34 (71)	309 (77)	0.306
Child–Pugh class A	42 (87.5)	337 (84.5)	0.58
Ascites history ^2^	6/47 (12.8)	54 (13.5)	0.568
Variceal bleeding history ^2^	5/47 (10.6)	31 (7.8)	0.495
NUCs (entecavir) ^2^	41 (85)	318 (80)	0.347

HBeAg, hepatitis B e antigen; AST, aspartate aminotransferase; ALT, alanine aminotransferase; AFP, α-fetoprotein; NUC, nucleos(t)ide analogue. ^1^ median (interquartile range); ^2^ no. (%).

**Table 2:** Predictors of hepatocellular carcinoma according to univariate and multivariate Cox regression models.

	Univariate Model		Multivariate Model	
Parameters	HR (95% CI)	*p*-Value	HR (95% CI)	*p*-Value
Age (years) (≥50 vs. <50)	2.17 (1.08–4.36)	0.03	2.34 (1.08–5.1)	0.032
Sex (male vs. female)	1.69 (0.79–3.62)	0.175		
Body mass index (kg/m^2^)	0.93 (0.86–1.0)	0.056		
HBeAg (positive vs. negative)	1.53 (0.72–3.27)	0.273		
HBV DNA (×10^5^ IU/mL) (≥4 vs. <4)	1.96 (1.03–3.74)	0.004		
Albumin (g/dL) (<3 vs. ≥3)	2.91 (1.04–8.15)	0.042		
AST (U/L)	1.0 (0.99–1.0)	0.26		
ALT (U/L)	1.0 (0.99–1.0)	0.268		
Total bilirubin (mg/dL)	0.99 (0.8–1.22)	0.9		
AFP (ng/mL) (≥8 vs. <8)	2.29 (1.28–4.1)	0.005	2.05 (1.1–3.83)	0.025
Hemoglobin level (g/dL)	1.0 (0.81–1.24)	1.0		
Platelet count (×10^3^/μL)	1.0 (0.99–1.0)	0.22		
Creatinine (mg/dL)	0.91 (0.53–1.55)	0.73		
Estimated GFR (MDRD)	1.0 (0.99–1.0)	0.16		
Fatty liver (yes vs. no)	1.8 (0.92–3.53)	0.089		
Splenomegaly (yes vs. no)	0.72 (0.38–1.34)	0.3		
Child–Pugh class A	0.98 (0.42–2.3)	0.96		
Ascites (yes vs. no)	1.14 (0.48–2.41)	0.762		
Variceal bleeding (yes vs. no)	1.52 (0.6–3.84)	0.38		

HR, hazard ratio; CI, confidence interval; HBeAg, hepatitis B e antigen; AST, aspartate aminotransferase; ALT, alanine aminotransferase; AFP, α-fetoprotein; GFR, glomerular filtration rate.

**Table 3:** Incidence rates of hepatocellular carcinoma stratified according to risk factors.

Hepatocellular Carcinoma	Events (*n*)	Observation Period	Rate/100 Person-Years	*p*-Value
Risk Factors		(Person-Years)	(95% CI)	
All	48	2352.8	2.04 (1.52–2.68)	
Age ≥50 years and AFP ≥8 ng/mL	21	669.2	3.14 (1.99–4.72)	0.004
Age ≥50 years and AFP <8 ng/mL	17	841.8	2.02 (1.22–3.17)	0.022
Age <50 years and AFP ≥8 ng/mL	8	287.9	2.78 (1.29–5.28)	0.01
Age <50 years and AFP <8 ng/mL	2	553.9	0.36 (0.06–1.19)	

AFP, α-fetoprotein; CI, confidence interval.

**Table 4:** Incidence rates of hepatocellular carcinoma stratified according age and HBV DNA.

Hepatocellular Carcinoma	Events (*n*)	Observation Period	Rate/100 Person-Years	*p*-Value
Risk Factors		(Person-Years)	(95% CI)	
All	48	2352.8	2.04 (1.52–2.68)	
Age ≥50 years and HBV DNA ≥4 × 10^5^ IU/mL	25	873.6	2.86 (1.89–4.16)	0.024
Age ≥50 years and HBV DNA <4 × 10^5^ IU/mL	13	637.4	2.04 (1.13–3.4)	0.058
Age <50 years and HBV DNA ≥4 × 10^5^ IU/mL	9	486.9	1.84 (0.9–3.39)	0.076
Age <50 years and HBV DNA <4 × 10^5^ IU/mL	1	354.9	0.28 (0.01–1.39)	

HBV, hepatitis B virus; IU, international unit; CI, confidence interval.

## Data Availability

The datasets used and/or analyzed during the current study are available from the corresponding author upon reasonable request.
